# Age of asthma onset and vulnerability to ambient air pollution: an observational population-based study of adults from Southern Taiwan

**DOI:** 10.1186/s12890-016-0218-0

**Published:** 2016-04-19

**Authors:** Tsung-Ju Wu, Chang-Fu Wu, Bing-Yu Chen, Yungling Leo Lee, Yue Leon Guo

**Affiliations:** Institute of Occupational Medicine and Industrial Hygiene, National Taiwan University, 17, Syujhou Road, Taipei, 100 Taiwan; Division of Chest Medicine, Department of Internal Medicine, Kaohsiung Municipal Min-Sheng Hospital, Kaohsiung, Taiwan; Department of Environmental and Occupational Medicine, National Taiwan University (NTU) College of Medicine and NTU Hospital, Taipei, Taiwan; Institute of Epidemiology and Preventive Medicine, National Taiwan University, 17, Syujhou Road, Taipei, 100 Taiwan; National Institute of Environmental Health Sciences, National Health Research Institutes, Zhunan, Taiwan

**Keywords:** Adult asthma, Air pollution, Particulate matter, Phenotype

## Abstract

**Background:**

Late-onset asthma (onset > 12 years) is pathologically distinct from early-onset asthma. The mechanism of air pollution is not a classic allergic inflammation and could have differential effect on late-onset and early-onset asthma. However, there is little known about the association of onset-age phenotype and air pollution. In this population-based study, we aimed to determine the association of asthma severity outcomes and air pollution regarding age at onset of asthma.

**Methods:**

In 2004, we conducted a cross-sectional questionnaire survey about respiratory health among schoolchildren’s parents randomly selected from 94 of 816 elementary and middle schools in southern Taiwan. Participants ever having typical asthma symptoms were enrolled. We used kriging method to estimate individual exposure to ambient air pollution in the preceding year before the year of asthma severity survey. Ordered logistic regression was used to determine the association of exposure and asthma severity scores. Age at asthma onset of 12 years was used as a cut-off to define early- or late-onset asthma.

**Results:**

The study surveyed 35,682 participants. Data from 23,551 participants remained satisfactory with a response rate of 66 %. Among 20,508 participants aged 26–50 years, 703 questionnaire-determined asthmatics were identified and included for analysis. Using the median of PM_10_ (66 μg/m^3^) as a cut-off, those exposed to higher PM_10_ were more likely to have higher severity scores (OR = 1.74; 95 % CI, 1.13 – 2.70) only for asthmatics with asthma onset at > 12 years.

**Conclusions:**

In adulthood, exposure to PM_10_ has a greater effect on late-onset asthma than early-onset asthma and deserves greater attention among ambient air pollutants.

**Electronic supplementary material:**

The online version of this article (doi:10.1186/s12890-016-0218-0) contains supplementary material, which is available to authorized users.

## Background

The association of diversity of asthma endpoints with air pollutants has been investigated for decades [1]. The acute effect of particulate matter (PM) on asthma exacerbation, indicated by increased asthma symptoms, medication use or healthcare use after a several-day lag of PM exposure has been observed both in childhood and adult asthma [2–5]. Diesel exhaust particulate, as a model of particulate pollutant, has a synergic effect to common environmental allergens [6]. Moreover, exposure to PM alone can induce airway hyperresponsiveness in the absence of allergic sensitization [7, 8]. Late-onset asthma is pathologically distinct from early-onset asthma and characteristic of differential disease outcomes in clinic-based and epidemiological studies [9, 10]. However, there is little known regarding the impacts of age at asthma onset on the response to air pollutants.

Two commonly used clinical practice guidelines, namely, National Asthma Education and Prevention Program guidelines and Global Initiative for Asthma guidelines, employ the use of rescue short-acting β2-agonist (SABA) and other medications to maintain control as the determinants of asthma severity [11, 12]. Along with these severity definitions, the inclusion of healthcare use to account for severe asthma was suggested by several other groups [13–15]. On the other hand, Eisner and collogues reported that a composite of scores for asthma severity, including SABA use, inhaled corticosteroid (ICS) use, oral medication use, hospitalization and respiratory symptoms, is able to predict clinical outcome of asthma without pulmonary function testing [16]. Therefore, a composite score could be suitable for evaluation of asthma severity in a large-scale population-based survey.

Adolescent-onset asthma was shown to be associated with more eosinophilic airway inflammation [17]. In addition, using age of 12 years as a cut-off was reported to differentiate an atopic and allergic phenotype from a more eosinophilic later-onset phenotype [9] and different risk factors [18]. As chronic residential traffic pollution was associated with eosinophilic airway inflammation in older asthmatics [19], we hypothesized that chronic exposure to air pollution has differential effect between early-onset (onset ≤ 12 years) and late-onset asthma (onset > 12 years). Kriging method has been validated as a reliable method for the estimation of air pollution exposure [20]. We tested the hypothesis in a population-based study by using kriging method for better classification of personal ambient air pollution exposure.

## Methods

In 2004, we conducted a school-based cross-sectional study including survey of respiratory diseases and symptoms for schoolchildren and their parents in southern Taiwan [21]. The population and territory of this area were 5,501,747 people and 7,914 square kilometers. Twenty of the 189 middle schools and 74 of the 627 elementary schools were randomly selected in proportion to the adult population in each county for investigation. Stratified sampling of candidate participants from each grade was conducted in each school. The questionnaire was sent through schoolchildren to their parents. A Chinese version of questionnaire modified from the questionnaire of the American Thoracic Society and the Division of Lung Diseases (ATS-DLD-78) was used to collect the related health information [22, 23]. The study protocol was approved by the Institutional Review Board (Human Study Committee) at the National Cheng Kung University Hospital. Each participant provided the written informed consent.

The study surveyed the parents of 35,682 children. A total of 12,131 subjects were excluded from our study due to inadequate demographic information or missing responses to the key questions. Data from 23,551 (66 %) subjects remained satisfactory. We enrolled the subjects aged 26–50 years as those who aged 26 years were the youngest parents in the sample and those who aged over 50 years might be confounded by chronic obstructive pulmonary disease and heart failure [24, 25]. Among 20,508 subjects aged 26–50 years, there were 703 participants with positive responses to questions used to identify asthma.

**Fig. 1:**
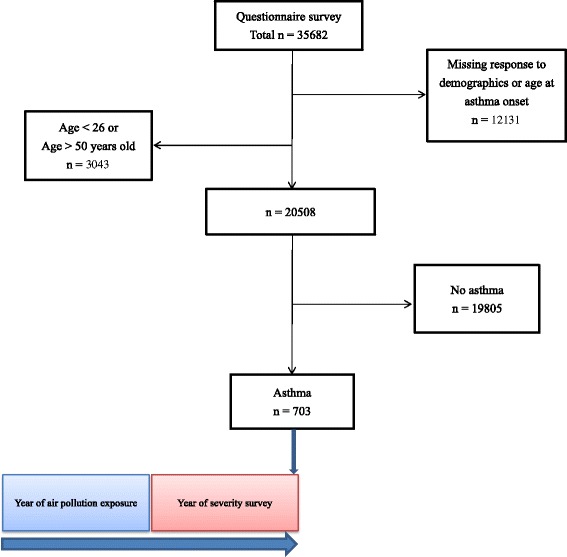
The flow chart describing the enrollment of study subjects and follow-up approach

The aim of this study was to evaluate the impact of air pollution on asthma severity and age of asthma onset. This observational study used a longitudinal approach in terms of the exposure in the preceding year to predict asthma severity in the following year (Fig. 1). As hospitalization and emergency department visits due to asthma attack were associated with ambient particulate matter in previous publication (estimated odds ratio = 1.7 by median of PM_10_) [26], the minimum sample size to reach 90 % of power and 0.05 of significance level for PM_10_ was calculated accordingly. The sample size to discriminate asthma severity outcomes by PM_10_ levels was calculated by proportion of 0.63 and 0.37 for exposed and non-exposed groups, respectively. As a result, the sample must include 168 asthmatics. Thus the reported number of asthmatics in this study was considered adequate for testing our hypothesis.

We developed a composite score modified by Eisner’s method for evaluation of asthma severity. Because we intended to evaluate asthma severity and chronic exposure to environmental factors, we extended the period of medication usage and health outcome to the preceding 12 months. On the other hand, for a better response rate in a large-scale population-based survey, we did not include detailed medication items as Eisner’s score. Thus, among 13 items of Eisner’s score, we included comparable items, namely frequencies of SABA use, ICS use, oral medication use, asthma attack, emergency department visit for asthma and hospitalization for asthma, but for the preceding 12 months to develop a modified Eisner score (Additional file 1: Table S1).

Measurement of air pollutants was obtained from air monitoring data routinely collected from 26 Taiwan Environmental Protection Administration air quality monitor stations in or surrounding southern Taiwan (Additional file 1). Another 15 assumed points located in the sea, 105 km from the coast, which is approximately half the distance between Taiwan and China, were added in the model as the background air monitoring stations. The southernmost station in Taiwan, i.e., Hengchun station, was a background monitoring station, and the air pollutant concentrations of the 15 assumed points were deduced from the levels of Hengchun station [27]. As aerosols under planetary boundary layer are well-mixed, subjects whose schools (4/94, 4 %) located over the elevation of 600 meters (the lower limit of planetary boundary layer in Taiwan) were excluded [28].

All stations in southern Taiwan are situated close to residential or industrial areas, except the one in Hengchun, the data of which was used as background values in kriging. Since the locations of the monitoring stations were not random, our sampling scheme was not drawn according to air monitoring stations. Kriging is a statistical mapping technique by which the prediction of unknown values over a spatial region was calculated from data collected at point locations. We used ArcGIS (ArcMap, version 10.0; ESRI Inc., Redlands, CA, USA) to perform kriging for estimating the yearly mean pollutant concentration at each school (Additional file 1). We evaluated the quality of the predicted values by the cross-validation – a procedure omitting data points once at a time and estimate the value at the omitting point with the remaining data. The measured concentrations at the ambient monitoring sites were then compared with the values predicted by kriging method.

### Statistical analysis

A composite score for asthma severity was assigned to each asthmatic, and the influence of air pollutants on the composite score was determined by ordered logistic regression with adjustment for demographic factors including age, sex, BMI, family income level and education level. An interaction term between “air pollutant level by median” and “age at onset by 12 years” was used to test whether asthma severity associated with air pollution varies according to age at onset. The asthmatics were then stratified by age at onset of 12 years for further analysis [9, 18]. As shown in previous documentations, air pollutants are not independent of each other. To examine the effect of copollutants, we used two-pollutant model to examine whether the observed effect of a single pollutant was actually attributed to another pollutant [29, 30]. The errors in the equations for school members are not independent because of the common school effect. Accounting for the clustered sampling scheme inherent in school member data, we used Huber's method to estimate robust standard errors to avoid an understatement of standard errors (Additional file 1). Stata 14.0 software (StataCorp LP, College Station, Texas) was used for analyses.

## Results

Among 703 enrolled asthmatics, late-onset asthma (onset > 12 years) was significantly associated with lower family income level. Late-onset asthma (onset > 12 years) was significantly associated with lower family income level. There was no significant difference between older onset group and younger onset regarding sex, age, BMI, education level and smoking status (Table 1).

**Table 1:** Characteristics of the study subjects (*N* = 703)

	Asthma onset > 12 years, n (%)		
	No	Yes	*P*
	(*n* = 253)	(*n* = 450)	
Age, years	38.8 ± 5.1	38.3 ± 4.8	0.2
Sex			0.1
Male	100 (39.5)	152 (33.8)	
Female	153 (60.5)	298 (66.2)	
BMI	23.8 ± 3.8	23.8 ± 4.0	0.9
Education			
Middle school or less	62 (24.5)	117 (26.0)	0.3
High school	119 (47.0)	228 (50.7)	
College or beyond	72 (28.5)	105 (23.3)	
Family income			0.02
< 13,300 USD	75 (29.6)	180 (40.0)	
13,300–33,000 USD	147 (58.1)	228 (50.7)	
> 33,000 USD	31 (12.3)	42 (9.3)	
Current smoking	62 (25.6)	96 (21.9)	0.3

*BMI* body mass index

Regarding current smoking status, the total numbers for asthmatics with age at onset ≤ 12 years and > 12 years were 242 and 438, respectively, because of missing data

**Fig. 2:**
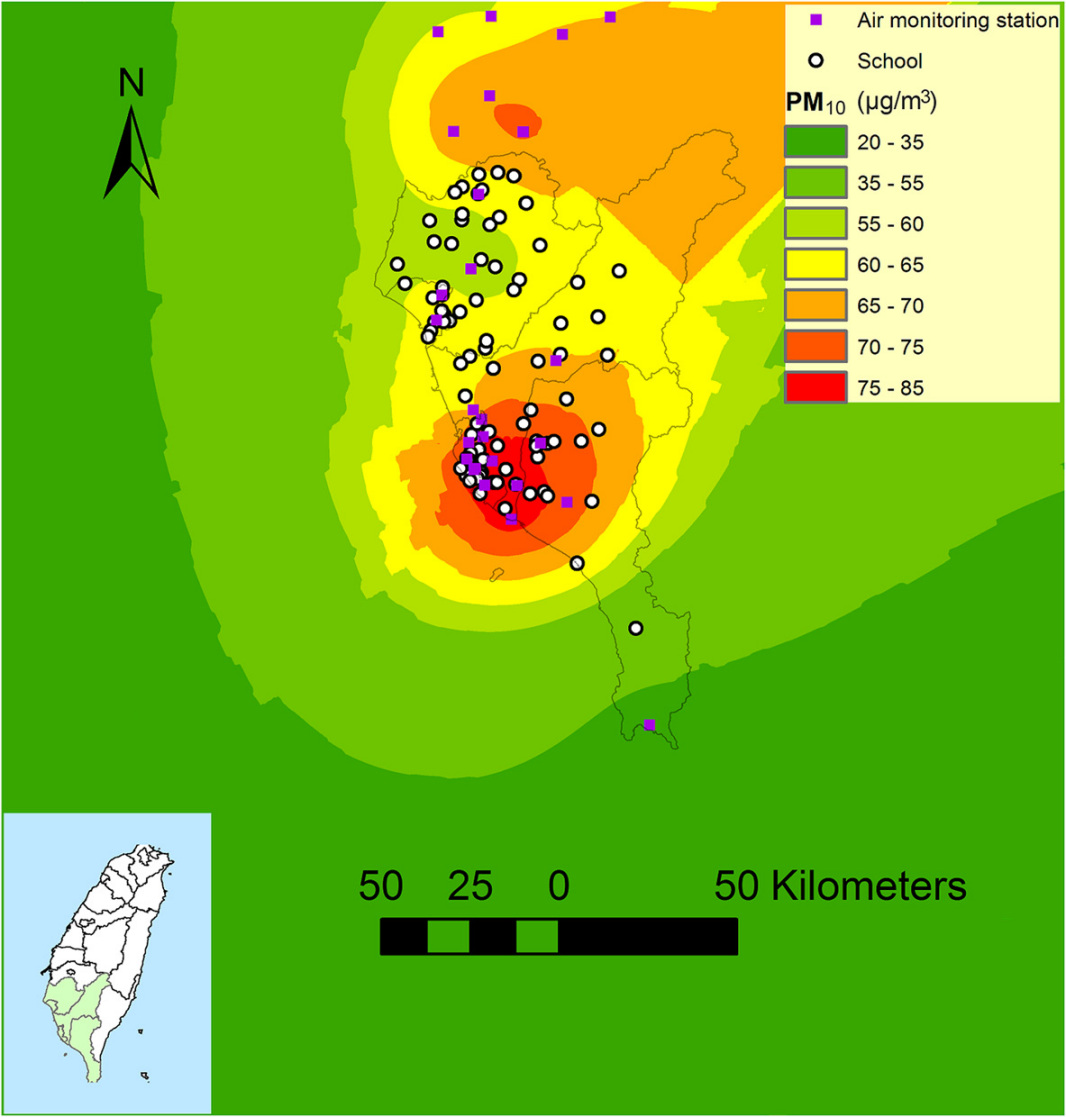
An example of estimated PM_10_ levels in southern Taiwan in 2003 by using air monitoring station data and kriging method. The schools located over the elevation of 600 meter were excluded

Figure 2 shows an example of estimated PM_10_ concentrations in 2003, air monitoring stations and sampled schools in southern Taiwan.

**Fig. 3:**
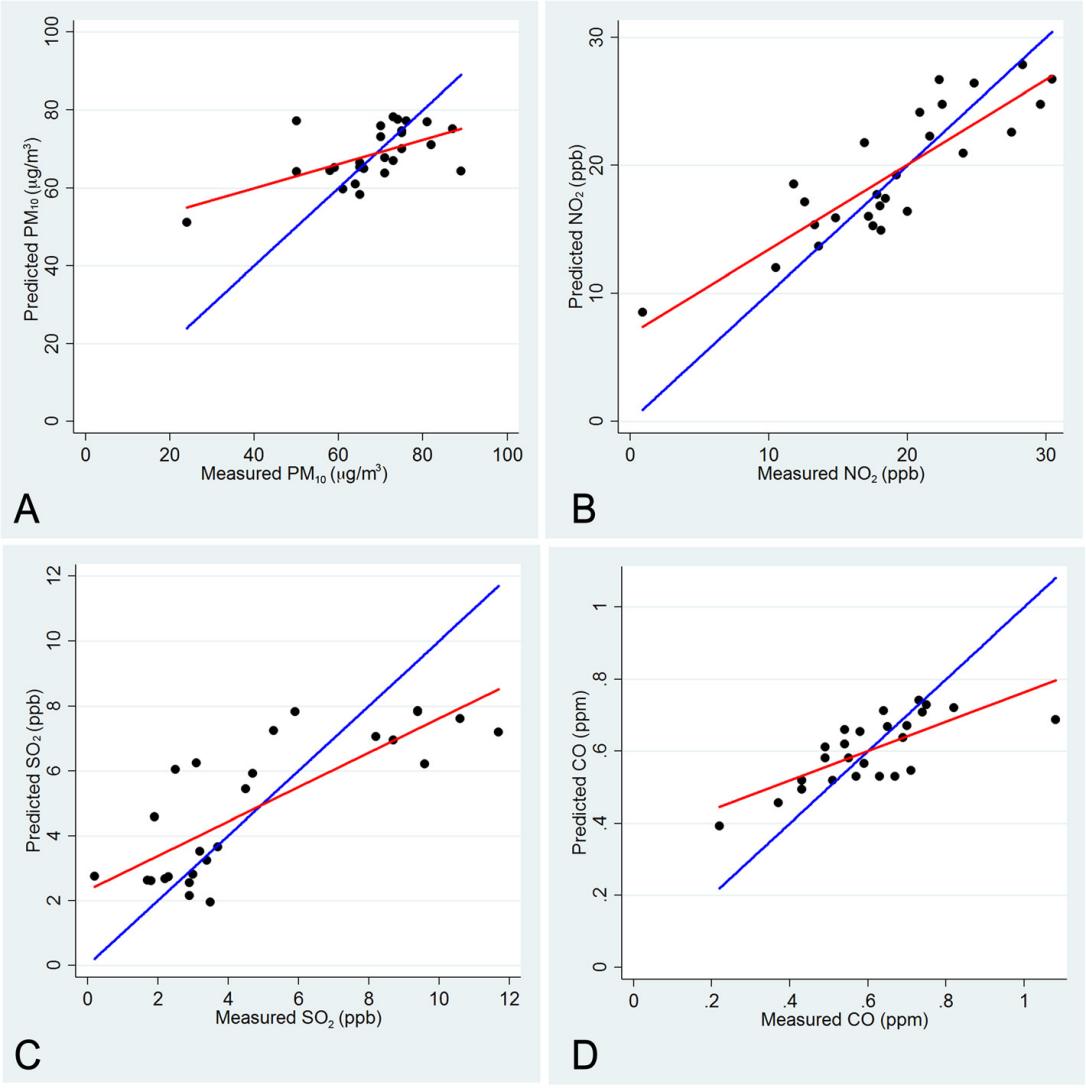
Plots of measured values and predicted values of PM_10_ (a), NO_2_ (b), SO_2_ (c), and CO (d) at air monitoring stations in 2003 by the kriging method. Regression lines and identity lines are shown in red and blue, respectively

The interquartile range was 61.0 to 71.0 μg/m^3^ for PM_10_, 16.9 to 23.6 ppb for NO_2_, 3.8 to 7.4 ppb for SO_2_, and 0.55 to 0.71 ppm for CO, respectively. The correlation of air pollutant values was relatively high with each other (*R* > 0.80) (Additional file 1: Table S2). The ratios of the geometric means of predicted air pollutant values over measured ones were 1.03 for PM_10_, 1.11 for NO_2_, 1.18 for SO_2_, 1.03 for CO in 2003, 1.02 for PM_10_, 1.10 for NO_2_, 1.17 for SO_2_, 1.03 for CO in 2002, respectively. The correlation coefficients of predicted and measured air pollutant values were 0.58 for PM_10_, 0.86 for NO_2_, 0.79 for SO_2_, 0.73 for CO in 2003, respectively (Fig. 3). Similarly, the correlation coefficients of predicted and measured air pollutant levels were 0.55 for PM_10_, 0.90 for NO_2_, 0.80 for SO_2_, 0.65 for CO in 2002, respectively. The result showed that the predicting model is a reasonable model for PM_10_, NO_2_, SO_2_ and CO. However, the correlation coefficients of predicted O_3_ and measured O_3_ were not high (*R* < 0.28). Therefore, O_3_ was not included in subsequent analyses.

The proportional odds assumption of the ordered logistic regression was verified and not violated (*p* = 0.07). Current smoking was not significantly associated with asthma severity score (OR, 1.00; 95 % CI, 0.65 ~ 1.52). Thus smoking status was not included in subsequent analyses. In the single pollutant model, by using the median of air pollutant concentration as a cut-off, those exposed to higher air pollutant levels were not associated with higher severity scores. However, the interaction term between “PM_10_ level by median” and “age at onset by 12 years” was significant (*p* = 0.03, Table 2). When the asthmatics were stratified by age at asthma onset of 12 years, those exposed to higher PM_10_ were more likely to have higher severity scores (OR = 1.74; 95 % CI, 1.13 – 2.70) only for asthmatics whose onset age was more than 12 years (Table 2). In two pollutant models, the effect of 1-year average of PM_10_ remained significant after adjusting for other pollutants (Table 3).

**Table 2:** The associations of chronic exposure to air pollutants and asthma severity stratified by age at asthma onset of 12 years

	All		Onset age > 12 years		Onset age ≤ 12 years		
	(*n* = 645)		(*n* = 413)		(*n* = 232)		
Severity score	aOR	95 % CI	aOR	95 % CI	aOR	95 % CI	*P* for interaction
PM_10_ > 66.0 μg/m^3^	1.37	0.93 – 2.00	1.74	1.13 – 2.70	0.87	0.49 – 1.55	0.03
NO_2_ > 18.5 ppb	1.15	0.77 – 1.72	1.29	0.81 – 2.04	0.92	0.49 – 1.71	0.28
SO_2_ > 4.5 ppb	1.30	0.86 – 1.95	1.52	0.95 – 2.43	1.00	0.54 – 1.83	0.19
CO > 0.61 ppm	1.13	0.75 – 1.69	1.21	0.76 – 1.92	1.03	0.56 – 1.90	0.54

*aOR* adjusted odds ratio; *BMI* body mass index; *CI* confidence interval

The odds ratios were adjusted with age, sex, BMI, education level and family income level. Those exposed to the pollutant concentrations lower than medians were used as reference groups. Numbers did not total 703 because of missing data. *P* values were for interaction terms between “air pollutant level by median” and “age at onset by 12 years”

**Table 3:** Two pollutant models for the associations of asthma severity and different air pollutants for asthmatics with asthma onset at > 12 years (*N* = 413)

	PM_10_ with NO_2_, aOR (95 % CI)	PM_10_ with SO_2_, aOR (95 % CI)	PM_10_ with CO, aOR (95 % CI)
Severity score	2.06 (1.02 – 4.18)	2.30 (1.01 – 5.21)	3.20 (1.52 – 6.73)

*aOR* adjusted odds ratio; *BMI* body mass index; *CI* confidence interval

The odds ratios were adjusted with age, sex, BMI, education level and family income level. Those exposed to the pollutant concentrations lower than medians were used as reference groups. Numbers did not total 450 because of missing data

## Discussion

In this study, we used a longitudinal follow-up approach, i.e., exposure to air pollutants in the preceding year and subsequent flare-up of asthma in the following year, to examine the chronic effects of air pollutants on severity of asthma in adulthood. We found that chronic exposure to PM is a risk factor for asthma severity in adulthood in late-onset asthma (onset > 12 years) but not in early-onset asthma (onset ≤ 12 years).

Asthma is a disease of chronic airway inflammation and airway hyperresponsiveness, which raise the plausibility that chronic exposure to PM could induce subsequent acute exacerbation of asthma. PM has been demonstrated to induce the formation of an excessive amount of reactive oxygen species in respiratory systems of experimental animals, leading to tissue inflammation and cell death [31]. In addition, there is evidence that ambient PM in a polluted urban environment could induce oxidative stress in humans [32]. As late-onset asthma (onset > 12 years) was characteristic with a less atopic status [9], the underlying pathophysiological mechanism might be a non-allergic mechanism or synergistic with allergic inflammation. Moreover, late-onset asthma might be associated with “non-T helper 2” phenotype, which age of asthma onset was reported to account for. The phenotype related to toll-like receptor and pathogen associated molecular pattern might explain the underlying non-atopic mechanism as well [33].

Previous literature has shown the association of emergency visits for asthma and air pollution both in adults and children [5, 34]. However, these reports did not distinguish early- and late-onset asthma as for the air pollutant effects. However, in our study, we observed the association of asthma severity and ambient PM_10_ only in those adults with late-onset asthma. The lacking of association between asthma severity and air pollutants among those adults with early-onset asthma deserves attention and further studies.

In previous literature, sufficient evidence infers a causal relationship between active smoking and exacerbation of asthma in adults [35]. However, such findings would require a longitudinal study design to be detected. In this current study, asthma severity score was not observed to be related to current smoking, likely because asthmatics who were susceptible to smoking tended to avoid smoking. As a result, the effect of current smoking on asthma severity was not observed in this study.

In our study, kriging method was used to predict the spatial distribution of the air pollutants, which utilized reliable pollution measurements from air monitoring stations throughout southern Taiwan to compute individual exposure estimates. In cases where many monitoring stations exist, kriging methods are often preferred to other interpolation methods, such as inverse distance weighted, spline, or global/local polynomials [36–38]. Therefore, the assignment of exposure by using yearly kriging from air monitoring stations should be one of the preferred methods. On the other hand, we used assumption background points in the sea to perform kriging to constrain estimates for known physical boundary conditions [39]. The PM distribution in our study was similar with the PM distribution in a study in southern Taiwan utilizing both inland and offshore sampling of air pollutants [40].

Though the correlation coefficients of measured and predicted values of PM_10_ were 0.55 in 2002 and 0.58 in 2003, respectively, we believed that these values were underestimated. The cross-validation method omitted data points one at a time and estimated the value at the omitted point with the remaining data. This procedure inevitably underestimates the value of the location with lowest pollutant and overestimates the value of the location with highest pollutant. Thus the correlation of PM_10_ would have been better between unmonitored locations and interpolated values than what was observed in the cross-validated models. Nevertheless, the regression line and identify line of measured and predicted values of PM_10_ were still relatively close. In addition, dichotomization of PM_10_ could attenuate the discrepancy between measured and predicted PM_10_. This was examined, and when the air pollutant levels were dichotomized, the correct predictive rates were 84.6 % for PM_10_, NO_2_, SO_2_ and 76.0 % for CO, respectively.

While we used the median of PM_10_ (66 μg/m^3^) as a cut-off, those exposed to PM_10_ higher than 66 μg/m^3^ were more likely to have higher severity scores than those exposed to PM_10_ lower than 66 μg/m^3^ (OR = 1.74; 95 % CI, 1.13 – 2.70). In other words, if the ambient PM_10_ concentrations were reduced to less than 66 μg/m^3^, an estimation of 30 % of asthmatics’ disease severity would have been decreased. Both in western and eastern countries, the incidences of adult-onset asthma have been reported to be increasing [41, 42]. In addition, a recent study showed that the prevalence of non-atopic asthma has been on the rise in adults [43]. Therefore, it would be important in the future to decrease severity of late-onset asthma by measures to reduce PM levels.

In our study, 34 % of participants was excluded due to missing demographics or the answer to the key question. However, among those with age and sex data, the distributions of age and sex were not statistically different between those included and those excluded in the final analysis. Thus the results would not have been distorted much.

Though we used a longitudinal approach to ascertain the time sequence of exposure and disease outcome, there is still a limit in determining the causal relationship based on this cross-sectional survey. In this current study, late-onset asthmatics who exposed to higher ambient PM_10_ were associated with higher severity scores. An alternative explanation of the observed association is that asthmatics who had higher severity scores would tend to live in area of higher PM_10_ level. Since the alternative explanation is less likely, the causal relationship would be relatively straightforward.

Our large cross-sectional questionnaire survey includes subjects in urban, suburban and rural areas in southern Taiwan, which enhance the representativeness of asthma patients in a general population. The personal variables were based on a questionnaire survey, which enable us to deal with many important confounders of asthma severity.

However, there are several limitations in our study. First, a cross-sectional study may not be well suited to identify onset age of asthma because onset age could have been mistaken. However, occurrence of asthma symptom is a crucial event and a cut-off age of 12 years corresponded to memorable personal histories (elementary school periods), which could lessen recall mistakes. Even in rare cases of recall mistakes, failure to remember early asthma history should not have affected current asthma severity. Therefore, such misclassification was unlikely to bias our main conclusion. Second, in this survey in southern Taiwan, only 55.5 % of subjects with typical symptoms had ever been recognized as having asthma by physicians [21]. Therefore the criteria of asthma were based on reported typical asthma symptoms, but without clinical confirmation in this study. Nevertheless, the questionnaire used in this study was the questions of American Thoracic Society and the Division of Lung Diseases (ATS-DLD-78) on asthma and asthma-like symptoms in adults, which have been validated [44] and has been widely used. Using these questions allows for comparisons with other epidemiological investigations. Third, for achieving a better response rate, we did not include the workplaces of the subjects in the questionnaire. In this study, we used ambient PM levels as the predictor, which could have been confounded by the indoor PM levels. Data from ambient monitors might not be an ideal surrogate for personal exposure estimation to total PM, as residence generated PM or occupational sources could not be accounted for in this study. However, the ambient PM concentration was highly correlated with personal exposures to ambient-generated PM, but not correlated with non-ambient PM [45]. Thus, residential or occupational PM is likely to result in nondifferential misclassifications of total personal exposure to PM, which could have reduced the observed associations towards the null. Since we still observed the association between ambient PM and severity for asthma, the relationship between PM_10_ exposure and asthma severity is likely present. Fourth, Large-scale studies on health effect of air pollution have been inevitably limited by an accurate measurement of personal air pollution exposure. In our study, we used air pollutant concentrations in schools as surrogates of homes. A report from the Taiwan Environmental Protection Administration showed that the coverage of the monitoring station was 3.3 km in radius for the PM_10_, 3.7 km for O_3_, 1.4 km for CO, 3.3 km for NO_2_, and 2.1 km for SO_2_, respectively, with a correlation coefficient of 0.9 [46]. Furthermore, in Taiwan, almost all children attended schools within 1 km of their homes. Therefore, we reasoned that air pollutant concentrations in schools provided reasonable indicators of home exposure.

## Conclusions

Chronic exposure to PM_10_ has a greater effect on late-onset asthma than early-onset asthma in adulthood and deserves greater attention among ambient air pollutants as the potential cause of increased severity of late-onset asthma. The differential effect of PM on early-onset and late-onset asthma highlights a specific pathophysiological mechanism of late-onset asthma. In previous publication, we reported a U-shape age distribution of asthma onset with a prominent second peak in the thirties in southern Taiwan [42]. As asthma is a complex disease composed of different disease variants, whether there is a distinct endotype prevalent in later-onset asthmatics in southern Taiwan warrants further investigation.

## Consent for publication

Not applicable.

## Availability of data and materials

The datasets of air pollution supporting the conclusions of the paper are available in the History Data Download repository of Taiwan Air Quality Monitoring Network (http://taqm.epa.gov.tw/taqm/tw/YearlyDataDownload.aspx). Due to the governance policy of confidentiality of the IRB, we can’t openly release the dataset of questionnaire survey underlying the conclusions of the paper available in public. A truncated dataset after eliminating all potentially identifiable features may be provided on an individual request basis.

## Additional file

Additional file 1: Table S1. Scoring for severity of asthma modified by Eisner’s method. Table S2: Pearson correlation coefficients of one-year average of air pollutants for asthmatics. (DOCX 23 kb)

## Abbreviations

ICS: inhaled corticosteroid
PM: particulate matter
PM_10_: particulate matter ≤ 10 μm
SABA: short-acting β2-agonist
