# Supplementary material for: Age of asthma onset and vulnerability to ambient air pollution: an observational population-based study of adults from Southern Taiwan
Source: BMC Pulm Med. 2016 Apr 19;16:54. doi: 10.1186/s12890-016-0218-0 (PMC4837625; doi:10.1186/s12890-016-0218-0)
Supplement: Additional file 1: Table S1. — Scoring for severity of asthma modified by Eisner’s method. Table S2: Pearson correlation coefficients of one-year average of air pollutants for asthmatics. (DOCX 23 kb) [file 12890_2016_218_MOESM1_ESM.docx]

**Age of asthma onset and vulnerability to ambient air pollution: an observational population-based study of adults from southern Taiwan**

Tsung-Ju Wu, Chang-Fu Wu, Bing-Yu Chen, Yungling Leo Lee, Yue Leon Guo

**Supplementary material**

**Kriging method**

Kriging is a statistical mapping technique by which the prediction of values over a spatial region was calculated from data collected at point locations. The value of the prediction location is formed by the measured values of nearby points weighted by distances from the prediction location and the degree of autocorrelation for those distances. Spatial autocorrelation of measured points was depicted by a semivariogram, in which a spatial model, such as spherical model in this study, was used to determine the parameters, namely partial sill, range and nugget [1-3].

**Estimation of the yearly mean pollutant concentration**

With respect to privacy, the participants’ exact addresses were not provided. Individual annual air pollutant levels between 2002 and 2003 were approximated according to the school addresses of their children. We used ordinary kriging method to construct the spatial exposure model. The air pollutant concentrations of the geocoded school-addresses were estimated by the air pollutant concentrations acquired from the Taiwan Environmental Protection Administration (EPA) monitoring stations in southern Taiwan. The one-year average of individual air pollutant level was calculated with a weighting factor of 0.5 and 0.5 for individual air pollutant value of 2002 and 2003, respectively.

**Measurement methods of air pollutants**

The data for PM_10_, O_3_, CO, NO_2_, and SO_2_ were obtained from the Taiwan EPA monitoring station. Briefly, PM_10_ was measured by beta-gauge, O_3_ was measured by ultraviolet absorption, CO by nondispersive infrared absorption, NO_2_ by chemiluminescence, and SO_2_ by ultraviolet fluorescence. The concentrations of air pollutant were measured continuously and reported hourly.

**Statistical model**

Asthma-severity*_ij_* = ß_0_+ ß_1_X*_i_* + ß_2_Z*_ij_* + δ*_i_* + μ*_ij_*

Asthma-severity*_ij_* is the severity of asthmatic *j* in school *i*. X*_i_* is a vector of air pollutant variables which are common to all school members (for example, PM_10_ and CO), and Z*_ij_* is a vector of variables which vary across all enrolled asthmatics (for example, sex, age, body mass index). The error term is assumed to be composed of one component which is common to all school members, δ*_i_*, and another component, μ*_ij_*, which varies independently across all enrolled asthmatics [4-5].

**References**

1. Cressie N. Spatial prediction and ordinary kriging. *Math Geol.* 1988;**20**:405-21.

2. Liao D, Peuquet DJ, Duan Y, Whitsel EA, Dou J, Smith RL, et al. GIS approaches for the estimation of residential-level ambient PM concentrations. *Environ Health Perspect.* 2006;**114**:1374-80.

3. Leem JH, Kaplan BM, Shim YK, Pohl HR, Gotway CA, Bullard SM, et al. Exposures to air pollutants during pregnancy and preterm delivery. *Environ Health Perspect.* 2006;**114**:905-10.

4. Huber PJ. The behavior of maximum likelihood estimates under non-standard conditions. In: *Proceedings of the Fifth Berkeley Symposium on Mathematical Statistics and Probability.* Berkeley: University of California Press; 1967. p. 221-33.

5. White H. A heteroskedasticity-consistent covariance matrix estimator and a direct test for heteroskedasticity. *Econometrica*. 1980;**48**:817-30.

Table S1. Scoring for severity of asthma modified by Eisner’s method

| Item No. | Description (during past year) | Scoring |
| --- | --- | --- |
| 1 | Symptoms (acute attack) | 0~4 |
|  | None | 0 |
|  | 1/year | 1 |
|  | 2~5/year | 2 |
|  | 6~10/year | 3 |
|  | > 10/year | 4 |
| 2 | Oral medication use | 0~4 |
|  | None | 0 |
|  | Occasional | 2 |
|  | ≥ 3 months/year | 4 |
| 3 | SABA use | 0~2 |
|  | None | 0 |
|  | Minimal | 0.5 |
|  | ≤ 1/month | 1 |
|  | > 1/month, ≤ 1/week | 1.5 |
|  | > 1/week | 2 |
| 4 | ICS use | 0~2 |
|  | None | 0 |
|  | Occasional | 1 |
|  | ≥ 3 months/year | 2 |
| 5 | Ever visiting emergency department for asthma | 0~4 |
|  | None | 0 |
|  | 1/year | 1 |
|  | 2/year | 2 |
|  | 3~5/year | 3 |
|  | ≥ 6/year | 4 |
| 6 | Ever hospitalized for asthma | 0~4 |
|  | None | 0 |
|  | 1/year | 1 |
|  | 2/year | 2 |
|  | 3~5/year | 3 |
|  | ≥ 6/year | 4 |
|  | Total possible score | 20 |

ICS: inhaled corticosteroid; SABA: short-acting β2-agonist.

Table S2. Pearson correlation coefficients of one-year average of

air pollutants for asthmatics (N = 703).

|  | PM_10_ | NO_2_ | SO_2_ | CO |
| --- | --- | --- | --- | --- |
| PM_10_ | 1.00 |  |  |  |
| NO_2_ | 0.81 | 1.00 |  |  |
| SO_2_ | 0.80 | 0.91 | 1.00 |  |
| CO | 0.86 | 0.95 | 0.95 | 1.00 |
